# Kératose pilaire

**DOI:** 10.11604/pamj.2019.33.274.16158

**Published:** 2019-07-30

**Authors:** Fatima-Zahra Agharbi

**Affiliations:** 1 Hôpital Civil Tétouan, Tétouan, Maroc

**Keywords:** Kératose, pilaire, atopie, kératolytiques, Keratosis, pilaris, atopy, keratolytics

## Abstract

Keratosis pilaris is characterized by small bumps around hair follicles. Keratosis pilaris simplex results in gray and keratotic papules mainly on the arms, thighs and buttocks. It mainly affects women. Keratosis pilaris rubra often results in keratosis pilaris atrophicans. Common variants of keratosis pilaris include: ulerythema ophryogenes, atrophoderma vermiculata, Siemens alopecia. Keratosis pilaris is a genetic disorder and can occur in association with other hereditary diseases such as Noonan syndrome or vitamin disorders. Treatment is mainly based on emollients and keratolytics, but they have only a suspensive effect. We report the case of a 30 year old woman with a personal and family history of atopy, presenting with diffuse keratotic papules on the trunk. The remainder of the physical examination revealed no genetic abnormality. Treatment was based on emollients and keratolytics with slight improvement.

## Image en médecine

La kératose pilaire est caractérisée par des petites élevures centrées sur les follicules pileux. La kératose pilaire simple est formée d'éléments gris et kératosiques sur les bras, les cuisses, les fesses principalement. Elle est plus fréquente chez les femmes. La kératose pilaire rouge a souvent une évolution atrophiante. Plusieurs tableaux sont réalisés: ulérythème ophryogène, atrophodermie vermiculée, alopécie de Siemens. Les kératoses pilaires sont essentiellement d'origine génétique et peuvent s'intégrer dans différentes maladies héréditaires, en particulier le syndrome de Noonan ou encore des troubles vitaminiques. Le traitement fait surtout appel aux émollients et aux kératolytiques, il n'est que suspensif. Nous rapportons l'observation d'une femme de 30 ans ayant comme antécédents une atopie personnelle et familiale qui consulte pour des papules kératosiques diffuses du tronc. Le reste de l'examen clinique ne trouvait pas de signes en faveur d'une anomalie génétique et la patiente a été mise sous kératolytiques et emollients avec légère amélioration.

**Figure 1:**
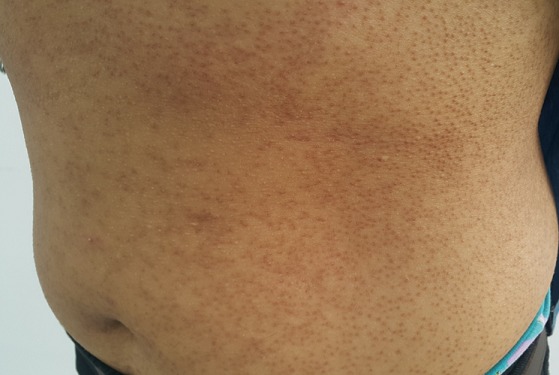
Papules kératosiques du tronc

